# Adult and elderly population access to trauma centers: an ecological analysis evaluating the relationship between injury-related mortality and geographic proximity in the United States in 2010

**DOI:** 10.1093/pubmed/fdx156

**Published:** 2017-11-28

**Authors:** B K Dodson, M Braswell, A P David, J S Young, L M Riccio, Y Kim, J F Calland

**Affiliations:** 1 Eastern Virginia Medical School, School of Medicine, Norfolk, VA, USA; 2 Institute for Advanced Studies in Culture, Charlottesville, VA, USA; 3 School of Medicine, University of Virginia, Charlottesville, VA, USA; 4 School of Medicine, Department of Surgery- Division of Acute Care Surgery and Outcomes Research, University of Virginia, Charlottesville, VA, USA; 5 Winchester Medical Center, Acute Care Emergency Surgery Services, Winchester, VA, USA

**Keywords:** emergency care, geography, mortality

## Abstract

**Background:**

Ongoing development and expansion of trauma centers in the United States necessitates empirical analysis of the effect of investment in such resources on population-level health outcomes.

**Methods:**

Multiple linear regressions were performed to predict state-level trauma-related mortality among adults and the elderly across 50 US states in 2010. The number of trauma centers per capita in each state and the percentage of each state’s population living within 45-min of a trauma center served as the key independent variables and injury-related mortality served as the dependent variable. All analyses were stratified by age (adult versus elderly; elderly ≥ 65 years old) and were performed in SPSS.

**Results:**

The proportion of a population with geographic proximity to a trauma center demonstrates a consistent inverse linear relationship to injury-related mortality. The relationship reliably retains its significance in models including demographic covariates. Interestingly, access to Levels I and II trauma centers demonstrates a stronger correlation with mortality than was observed with Level III centers.

**Conclusion:**

Trauma center access is associated with reduced trauma-related mortality among both adults and the elderly as measured by state reported mortality rates. Ongoing efforts to designate and verify new trauma centers, particularly in poorly-served ‘trauma deserts’, could lead to lower mortality for large populations.

## Background

Traumatic injury is the leading cause of death for all Americans between 1 and 44 years of age, and is responsible for ~20% of all life years lost before the age of 85 in the United States.^1,2^ Most of the US operates under an inclusive trauma center designation and verification model whereby hospitals are designated by a given state’s department of health and verified by site-inspectors from the same (or similar) state agency, or another group such as the American College of Surgeons. In general, the capabilities of each center are verified as falling between levels I and IV according to widely accepted national guidelines that define the highest level of trauma care capability as Level I. The most severely injured victims are typically transferred directly to a Level I trauma center if one is available in reasonable geographic proximity to where the injury occurred, or, otherwise, to a lower level center that is closer.^3^

Empirical investigation of the effect of trauma center access is necessary in light of the growing number of trauma centers and the lingering discrepancies in the types of trauma centers that predominate in different areas, i.e. rural versus urban. Additionally, ongoing increases in the proportion of the American population classified as elderly underscore the importance of their inclusion in analyses of this kind, especially considering the fact that some studies have suggested elderly patients may benefit less from access to advanced trauma care than their adult counterparts.^4–6^ This study offers an initial assessment of the impact of trauma center access on population-level trauma-related mortality. Given the known evidence that treatment at trauma centers is superior to non-trauma centers with respect to mortality, we hypothesized that injury-related mortality would be lower in states with greater access to advanced trauma care.

## Methods

This retrospective, ecological analysis uses IBM SPSS 20 software (Chicago, IL) to construct simple and multivariate linear regression models to determine the association between senior and adult population access to designated trauma centers (DTCs) (‘trauma center access’) in each state and their respective crude injury-related mortality rates. Each of the 50 states represented a unique case in our data set; the District of Columbia was excluded as a statistical outlier given its unusually large number of trauma centers per capita (data not shown). In an attempt to account for the unequal distribution of the national populace, the states were weighted to accurately reflect their representation with respect to the total population, meaning ‘trauma center access’ in populous states will have a greater impact on the results than access in less populous states.^7^ Association of ‘trauma center access’ and injury-related mortality was defined as statistically significant linear regression standardized coefficient (*β*). Adjusted *R*^2^ values were also used to demonstrate the variability in the mortality that is explained by the changes in the independent variable, which is ‘trauma center access’ (as defined below). The University of Virginia Institutional Review Board deemed the project as exempt from requiring approval as individual patient records were not accessed and only publicly accessible information was used.

Utilizing the Centers for Disease Control and Prevention Web-based Injury Statistics Query and Reporting System (CDC WISQARS), the crude injury-related mortality rate by state was determined by tallying all injury-related deaths per 100 000 people occurring in individuals 16–64 years of age (for the adult population) and 65 years of age and older (for the elderly population), including all injury intents, for all states in 2010.^7^ These mortality rates served as the dependent variables in all subsequent analyses. Two distinct sets of independent variables served as measures of ‘trauma center access’ and thus two separate analyses were performed to establish the relationship between ‘trauma center access’ and injury-related mortality. The first analysis utilized registries from the Trauma Information Exchange Program (TIEP) to determine the number of DTCs per 100 000 people in each state in 2008 (Hereafter called ‘per capita data’).^8^ Three separate independent variables were used in this analysis: Level I DTCs per capita; Level II DTCs per capita; and Level III DTCs per capita. In the second analysis the method of measuring ‘trauma center access’ was based upon the proportion of each state’s population living within 45 min by ground of a DTC in 2010 drawn from the University of Pennsylvania’s Trauma Maps data set (Hereafter called ‘proximity data’).^9^ In this analysis, three different independent variables were derived from the trauma maps data and were grouped as follows: percentage of each state’s population within 45 min by ground of a Level I DTC; percentage of each state’s population within 45 min by ground of a Level I or II DTC; and percentage of each state’s population within 45 min by ground of a Levels I, II or III DTC. For the elderly analyses, proximity to DTCs was calculated based upon the senior population only. For the adult analyses, proximity was determined using the total population, as proximity data for adults alone was not available. The decision to utilize the proximity variables based on ground transportation alone was rooted in the fact that weather and equipment issues may limit the degree to which air transportation can be depended upon.

In both analyses just described, the principal independent variables served as the sole independent variable in separate simple regression models to determine their relationship with mortality. Subsequently, covariates previously postulated to be predictors of injury-related mortality were then entered individually into our regression models to construct multivariate regression models which allowed us to assess the resilience of ‘trauma center access’ compared to these possible confounding variables (see below). Trauma center access was deemed to be an independent predictor of mortality if the relationship with injury-related mortality maintained statistical significance after the addition of the aforementioned variables. Because the number of cases in the data set is relatively small, only one covariate at a time was included in the regression models.

Determination of equal access to DTCs in the elderly and adult populations was ascertained using the independent *t*-test method in SPSS.

## Results

**Fig. 1:**
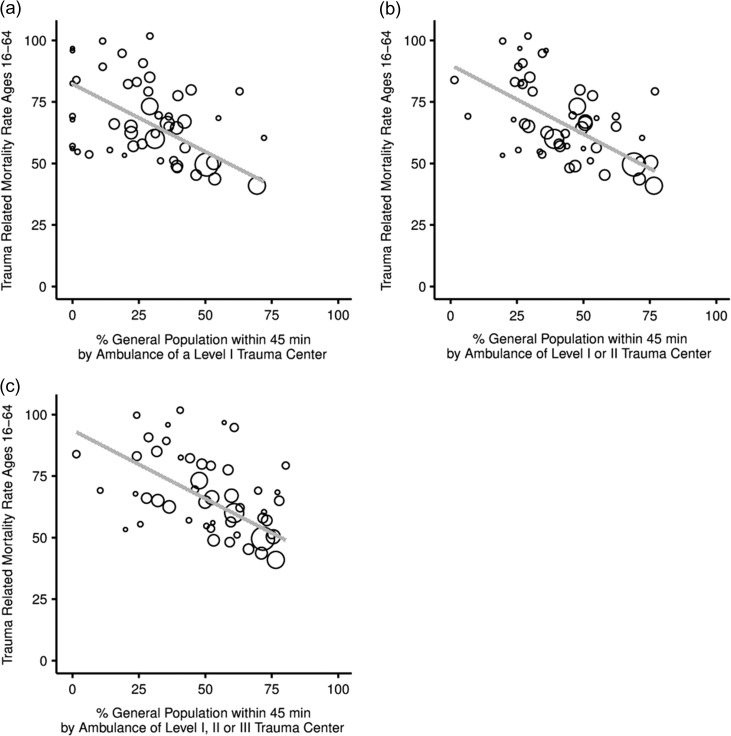
Scatterplots for adult. (a) total population proximity to Level I (2010) and adult injury-related mortality (2010); (b) total population proximity to Level I or II (2010) and adult analyses injury-related mortality (2010); and (c) total population proximity to Level I, II, or III (2010) and adult injury-related mortality (2010). (Larger Circles indicate more populous states.)

**Fig. 2:**
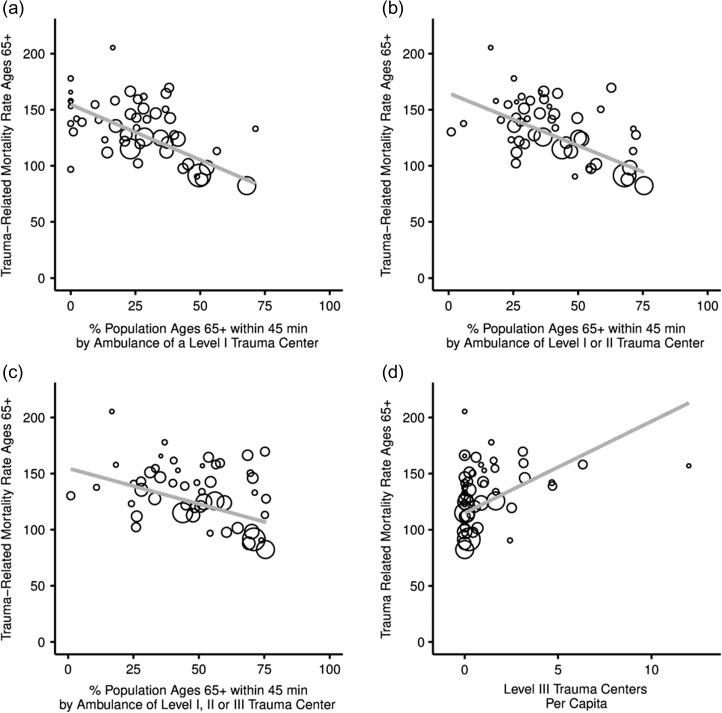
Scatterplots for elderly analyses. (a) Senior proximity to Level I (2010) and elderly injury-related mortality (2010); (b) proximity Level I or II (2010) and elderly injury-related mortality (2010); (c) proximity to Level I, II, or III (2010) and elderly injury-related mortality (2010); and (d) Level III trauma centers per capita (2008) and elderly injury-related mortality (2010).

Simple linear regression models and the associated graphical representations (scatterplots) demonstrate a statistically significant inverse relationship between proximity to DTCs and crude injury-related mortality rates (Figs 1a–c and 2a–c; Table 1, all rows; Tables 2 and 3, first rows). In simple regression models, proximity to a Level I or II trauma center appears to be the greatest predictor of mortality, both among adults (*P* < 0.001; *β* = −0.687; Adj. *R*^2^ = 0.460) and among the elderly (*P* < 0.001; *β* = −0.669; Adj. *R*^2^ = 0.437) (Tables 2 and 3, first rows). Access to Level I DTCs alone and Levels I, II and III DTCs in combination are also predictive of reduced mortality in the adult and elderly populations (Table 1).

**Table 1:** Simple regression outputs—relationship between percentage of adult and elderly population access to Level I alone and Level I, II or III combined within 45 min by ground in 2010 and injury-related mortality in 2010

Model	Independent variable	Standardized coefficient	*P*-value	Confidence intervals	Adj. *R*-squared
Simple	Adult population access to Level I Trauma Centers	−0.615	<0.001	−75.508 to −34.593	0.366
Simple	Elderly population access to Level I Trauma Centers	−0.654	<0.001	−131.724 to −65.512	0.416
Simple	Adult population access to Level I, II or III Trauma Centers	−0.657	<0.001	−74.260 to −37.145	0.420
Simple	Elderly population access to Level I, II or III Trauma Centers	−0.442	=0.001	−101.203 to −26.163	0.179

**Table 2:** Regression outputs—relationship between total population access to Level I or II trauma centers within 45 min by ground in 2010 and adult injury-related mortality in 2010

Model	Variable	Standardized coefficient	*P*-value	Confidence intervals	Adj. *R*-squared
Simple	Access to Level I or II Centers	−0.687	<0.001	−73.506 to −38.942	0.460
Bivariate with % African American (2010)^10^	Access to Level I or II Centers	−0.686	<0.001	−73.983 to −38.320	0.449
	% Af. Am.	0.004	0.967	−37.720 to 39.289	
Bivariate with % ages 18–44 (2010)^11^	Access to Level I or II Centers	−0.617	<0.001	−68.377 to −32.665	0.489
	% Ages 18–44	−0.208	0.061	−386.315–8.985	
Bivariate with % College-Education (2007)^12^	Access to Level I or II Centers	−0.299	0.011	−43.024 to −6.006	0.651
	% College-educated	−0.586	<0.001	−269.504 to −119.574	
Bivariate with GDP (current dollar, 2012)^13^	Access to Level I or II Centers	−0.575	<0.001	−67.342 to −26.978	0.490
	GDP	−0.214	0.087	−10.991 to 0.772	
Bivariate with % Non-White (2010)^14^	Access to Level I or II Centers	−0.663	<0.001	−72.897 to −35.733	0.453
	% Non-White	−0.067	0.557	−40.119 to 21.892	
Bivariate with population density (2010)^15^	Access to Level I or II Centers	−0.605	<0.001	−69.107 to −30.036	0.471
	Population density	−0.168	0.162	−0.024 to 0.004	
Bivariate with % uninsured (2012)^16^	Access to Level I or II Centers	−0.634	<0.001	−69.286 to −34.528	0.490
	% Uninsured	0.207	0.056	−2.082 to 153.604

**Table 3:** Regression outputs—relationship between percentage of the senior population with access to Level I or II Trauma Center within 45 min by ground in 2010 and injury-related mortality in 2010

Model	Variable	Standardized coefficient	*P*-value	Confidence intervals	Adj. *R*-Squared
Simple	Access to Level I or II trauma centers	−0.669	<0.001	−123.495 to −63.287	0.437
Bivariate with % African American (2010)^10^	Access to Level I or II trauma centers	−0.718	<0.001	−127.652 to −72.715	0.541
	% Af. Am.	−0.338	=0.001	−165.623 to −43.667	
Bivariate with % ages 65+ (2010)^11^	Access to Level I or II trauma centers	−0.673	<0.001	−124.588 to −63.232	0.425
	% Ages 65+	−.029	=0.795	−329.867 to 253.971	
Bivariate with % college-educated (2007)^12^	Access to Level I or II trauma centers	−0.765	<0.001	−147.550 to −65.763	0.436
	% College-educated	0.141	=0.339	−86.437 to 246.004	
Bivariate with GDP (current dollar, 2012)^13^	Access to Level I or II trauma centers	−0.481	<0.001	−100.632 to −33.060	0.522
	GDP	−0.347	=0.006	−25.193 to −4.433	
Bivariate with % Non-White (2010)^14^	Access to Level I or II trauma centers	−0.518	<0.001	−101.007 to −43.545	0.563
	% Non-White	−0.394	<0.001	−141.402 to −44.385	
Bivariate with population density (2010)^15^	Access to Level I or II trauma centers	−0.530	<0.001	−107.874 to −39.917	0.479
	Population density	−0.267	=0.032	−0.052 to −0.002	
Bivariate with % Uninsured (2012)^16^	Access to Level I or II trauma centers	−0.709	<0.001	−130.299 to −67.622	0.442
	% Uninsured	−0.136	=0.228	−225.651 to 55.141	

As evidenced in the adult and elderly multivariate regression models analyzing the association of trauma center access within 45 min by ground and mortality, access to Level I; Level I or II; and Level I, II or III DTC, appear to be independent predictors of injury-related mortality (multivariate regression analyses of the impact of proximity to Level I or II trauma centers are shown in Tables 2 and 3; the multivariate analyses containing Level I and Level I, II or III are not shown). In the adult cohort, educational attainment proves to be the only covariate capable of exhibiting a greater association with trauma-related mortality than the proximity variables, although the proximity variables retain their significance even in these models (Table 2). For the elderly, in contrast, educational attainment never approaches statistical significance in the proximity models (Table 3). Instead, race, GDP and population density serve as the most powerful predictors of mortality, although none as strong as proximity to DTCs (Table 3).

While the elderly population and the adult population had equal access to trauma centers (levene’s *F* = 0.069 and *P* = 0.793, mean 26.41 versus 28.10, SD 18.45 versus 19.1, (*t*)−0.451, df 98, two-tailed *P* = 0.653), the respective correlations with mortality followed different patterns. More of the variations in mortality for the elderly population appears to be explained by access to Level I DTCs as compared to the adult population (Elderly versus adult-Adj. *R*^2^ 0.416, *P* < 0.001 versus Adj. *R*^2^ 0.366, *P* < 0.001) whereas proximity to trauma centers in adult populations appears to explain more of the variations in mortality after the addition of Level II trauma centers into the model (elderly versus adult-Adj. *R*^2^ 0.437, *P* < 0.001 versus Adj. *R*^2^ 0.460, *P* < 0.001) than with Level I DTCs alone. When Level III centers are added to the model, very little of the variation in mortality is explained by proximity in the elderly population while adult proximity continues to account for much of the mortality trends. (elderly versus adult-Adj. *R*^2^ 0.179, *P* = 0.001 versus Adj. *R*^2^ 0.419, *P* < 0.001)

Simple regression models using trauma centers per capita as the independent variable did not reach statistical significance in the adult population for Level I, II or III DTCs. In the elderly cohort, neither Level I nor II trauma centers per capita were predictive of mortality; however, Level III DTCs per capita appear to be directly proportional to injury-related mortality, suggesting that states with greater numbers of Level III trauma centers per capita have higher rates of injury-related mortality in their elderly populations. (Fig. 2d, *P* = 0.002). This relationship held true in multivariate regression models indicating that Level III trauma centers per capita is an independent predictor of increased mortality in the elderly population (Table 4).

**Table 4:** Regression outputs—relationship between Level III trauma centers per capita in 2008 and elderly injury-related mortality in 2010

Model	Variable	Standardized coefficient	*P*-value	Confidence intervals	Adj. *R*-squared
Simple	Level III trauma centers per capita	0.425	=0.002	3.118 to 13.204	0.164
Bivariate with % African American (2010)^10^	Level III trauma centers per capita	0.392	=0.006	2.315 to 12.709	0.165
	% Af. Am.	−0.140	=0.305	−127.103 to 40.693	
Bivariate with % Ages 65+ (2010)^11^	Level III trauma centers per capita	0.445	=0.002	3.420 to 13.639	0.162
	% Ages 65+	0.127	=0.956	−185.589 to 521.576	
Bivariate with % College-Educated(2007)^12^	Level III trauma centers per capita	0.400	=0.002	2.948 to 12.398	0.271
	% College-educated	−0.347	=0.007	−336.245 to −56.893	
Bivariate with GDP (current dollar, 2012)^13^	Level III trauma centers per capita	0.316	=0.005	1.900 to 10.174	0.460
	GDP	−0.557	<0.001	−33.054 to −14.546	
Bivariate with % Non-White (2010)^14^	Level III trauma centers per capita	0.313	=0.007	1.719 to 10.305	0.422
	% Non-White	−0.507	<0.001	−176.738 to −71.308	
Bivariate with population density (2010)^15^	Level III trauma centers per capita	0.292	=0.019	0.955 to 10.265	0.348
	Population density	−0.460	<0.001	−0.071 to 0.022	
Bivariate with % Uninsured (2012)^16^	Level III trauma centers per capita	0.423	=0.002	3.024 to 13.213	0.149
	% Uninsured	0.054	=0.685	−132.243 to 199.704	

## Discussion

### Main finding of this study

Greater trauma center access, as defined by the percentage of each state's populace that lives within 45 min by ground of a designated trauma center, was associated with lower injury-related mortality rates in the adult and elderly populations. This relationship is true for models including Levels I, II and III trauma centers. Multivariate regression models provide proper adjustment for previously proposed predictors of mortality, including rurality, and thus account for the magnitude of confounding biases.

### What is already known on this topic

Abundant research indicates that care provided at DTCs is superior to the care provided at non-trauma centers as measured by outcomes, a difference that has been primarily linked to severely injured patients.^17–20^ Between 2005 and 2010, 21 Level I and 17 Level II trauma centers were added across the nation and by 2010, 90% of the US population had access to designated Level I or Level II trauma centers within 60 min by ground or air.^8,9^ However, large swaths of the American population still lack convenient access to the most advanced trauma center care. This is particularly true in rural states and regions, where non-tertiary trauma centers, i.e. Levels III and IV centers, often provide a large majority of the available trauma care.^21,22^

Previous research suggests that under-triage is a common problem for elderly patients and has been associated with increased mortality and increased costs.^23–26^ Under-triage could be a result of the masking effects of medication, high likelihood of co-morbid conditions, polypharmacy, unique mechanisms of injury, atypical presentation or likelihood for rapid decline.^27–30^ Innocenti *et al.*^29^ found that adults and elderly patients admitted with the same sequential organ failure assessment score (SOFA) and modified early warning scores (MEWS) had vastly different scores after 24 h with the elderly patient’s being notably higher. Under-triage can pose a major problem if the closest trauma center to the patient is a Level III DTC that may not be as equipped to handle severely injured patients after deterioration to the extent that a Level I or II center maintains clinical readiness. Additionally, elderly patients may be less tolerant of transfer times both at injury and after clinical decline at a Level III center.^26,31^

It has been well documented that social determinants of health affect outcomes after traumatic injury. Insurance coverage has been associated with reductions in complications, decreases in mortality, and more prevalent use of post-acute hospitalization resources in the adult population.^32–36^ The effect of race on injury-related mortality has been extensively studied; there are mixed results describing the impact of race on mortality, complications, functional outcomes and utilization of post-discharge resources, with all tending toward worse outcomes for minorities or no difference.^36,37^ One group found that treatment in hospitals with higher proportions of African American to white patients was correlated with higher mortality for patients of all races treated at that institution.^36^ Haider *et al*.^36^ performed a literature review and meta-analysis where the data allowed and found that the preponderance of available research demonstrates African American ethnicity to be associated with increased mortality compared to other ethnicities. The results for other non-white racial groups combined were inconclusive insofar as the data is lacking and inhibited the ability to perform meta-analyses.^36^

### What this study adds

Among adults, the finding that proximity to Level I and Level II trauma centers in combination yield a substantially higher adjusted *R*^2^ and standardized *β* than do Level I centers alone suggests the existence of real added benefit from Level II centers. However, given the nature of this population-level model, it is not possible to establish causality. The fact that including Level III centers in the independent variable yields a lower adjusted *R*^2^ and standardized coefficient than when the independent variable excludes Level III centers, along with the fact that Level III centers showed a positive (though not significant) relationship with mortality in the per capita analysis, could suggest that such centers do not necessarily exert the same type or magnitude of added benefit, for population mortality, compared to Levels I and II.

The inverse correlation between Levels I and II trauma center proximity and injury-related mortality in the elderly analysis lends evidence to the power of these specialized centers to influence the health of the senior population after traumatic injury. The fact that these results appear to be divergent from those of MacKenzie *et al.* who found that patients older than 55 have similar outcomes whether they were treated at a DTC or at a non-designated trauma centers (NTCs) could be due to the restrictive inclusion/exclusion criteria of their comparative study, their access to patient level records, or the fact that the current investigation does not include a direct comparison of the care provided at DTCs versus NTCs.^5^ Moreover, other authors have found that DTCs outperform NTCs even when caring for severely injured elderly patients.^18,20^

The weak and inconsistent correlation between mortality and senior population access to Level III DTCs in simple and multivariate regression modeling may be the result of the high likelihood for under-triage in the elderly population and not necessarily a direct representation of the care at those centers. Furthermore, elderly patients and their families may choose to de-escalate care given the seemingly insurmountable injury burden and their relative frailty which would impact trauma-related mortality values. Finally, elderly specific trauma triaging policies could potentially help in capturing severely injured senior citizens and improve expeditious transfer to appropriate definitive care which could improve trauma-related mortality in this cohort.^38^ On the other hand, elderly patients do remain at higher risk for major medical and surgical complications, have higher mortality rates (both in hospital and after discharge), greater average injury severity scores (ISS), higher mortality associated with lower ISS, increased likelihood of non-routine discharge and longer hospital stays than their adult counterparts, all of which have been attributed to the reduced functional capacity of organ systems, altered physiology, unique mechanism of injury, polypharmacy, increasing frailty and the higher incidence of medical co-morbidities.^6,28–30,39–48^ Studies investigating the age at which these differences take effect have been published: Goodmanson *et al.*^18^ found a significant increase in mortality starting at age 57 and Di *et al.*^17^ found differences in outcomes as early as 45. The complexity of trauma care in the elderly population could explain the differences among the adult and elderly populations, specifically the relatively low adjusted *R*-square in the proximity analyses including Level III trauma centers (Elderly versus Adult –0.179 versus 0.420) and the direct correlation between elderly mortality and Level III trauma centers per capita. The findings in this analysis could suggest less predictable outcomes for elderly patients treated at Level III trauma centers compared to elderly patients treated at Level I or II trauma centers and compared to adults treated at Level III trauma centers.

Insurance status appears to be less relevant in the elderly population, likely due to the near total coverage that this population enjoys under the US federal Medicare Program; these findings align with previous research that found no interrelationship.^49,50^ Singer *et al.* found that elderly patients are more likely to have insurance than adult trauma patients by a factor of four. Interestingly, they also discovered that elderly patients without insurance have similar mortality rates as those covered under Medicare.^50^ Of important note, elderly specific insurance data by state was not located to include in this analysis, which makes adjustment for the true number of senior citizens without insurance difficult to execute.

In the elderly analysis, the relationship between minority race and mortality is inversely proportional, meaning the higher the percentages of non-white people or African American people per state the lower the mortality. This result could suggest that the current analysis is really a measure of the diversity of the state rather than a test of the effect of race on injury-related mortality. Alternatively, the results could be interpreted to be in keeping with previous studies that found a reverse or reduction in the effect of racial disparities on trauma outcomes in the elderly.^50,51^

One of the most striking divergences between the adult proximity analyses and the elderly proximity analyses is the far greater impact of educational attainment on adult trauma-related mortality. Educational attainment is consistently shown to the most powerful predictor of mortality among adults, with an effect even greater than that of trauma center access. This is in stark contrast to the elderly analysis where the relationship between education and injury-related mortality does not approach significance. (It should be noted that the measure of educational attainment used in these analyses reflects 4-year college completion across the entire state population, with no age-based restrictions.) The difference in the effect is likely due at least in part to the fact that, as Dupre documented, differences in health between the highly-educated and the less-educated peak during mid-life and subsequently decline.^52^ It also seems likely that access to Medicare among the oldest Americans lessens the impact of an insurance coverage gap that would be linked to educational attainment. Ultimately, however, more research in this area is warranted.

### Limitations of this study

To the best of the authors’ knowledge, this study represents the only population-level representation of the relationship between DTCs and injury-related mortality in the elderly and adult populations. However, in attempting to extrapolate these findings to individual trauma victims, one must bear in mind that the specific circumstances surrounding each trauma case are not included in this analysis; this lends itself to ecological fallacy, which is inherent in analyses conducted at the population level. Furthermore, care across NTCs and non-tertiary trauma centers (levels III–IV) varies and cannot be assumed to be sub-optimal at either institution. It is conceivable that some centers are practicing within the specifications recommended by the American College of Surgeons but, have not been designated or verified and their presence would potentially skew the results. Detailed analysis investigating the relationship between injury-related mortality and appropriate advanced trauma care at the level of individual patient encounters is in order.

This analysis also does not allow for an evaluation of the relationship between individual trauma center levels independently and injury-related mortality in a model containing a distance metric, which is, in the authors’ opinion, paramount. Future research plans involve the use of these metrics to better support the suggestion that adult and elderly populations truly benefit from access to specialized trauma care and, more importantly, to determine if care at Level I centers provides mortality benefit over Level II and/or Level III. Additionally, it is important to note that this analysis does not take into account the injuries resulting in death on the scene before specialized trauma care could potentially prove beneficial; as has been demonstrated in the past, it is likely that injury mechanisms differ greatly amongst rural versus urban populations and their respective probabilities of instantaneous death could differ.^53^

## Conclusions

Among adults and seniors, access to Levels I and II DTCs is inversely correlated with injury-related mortality when incorporating a measure of distance. This research contributes to the justification for the expense involved in the maintenance of readiness at Levels I and II DTCs. Access to Level III DTC is predictive of reduced rates of injury-related mortality in the adult population which speaks to their capability in treating adult trauma patients. Care of the elderly at Level III trauma centers requires more investigation in light of the uncertain relationship between mortality and access to such centers, the high rates of under-triage, and the complex nature of trauma care in this patient population.
